# Enterovirus A71 2B Inhibits Interferon-Activated JAK/STAT Signaling by Inducing Caspase-3-Dependent Karyopherin-α1 Degradation

**DOI:** 10.3389/fmicb.2021.762869

**Published:** 2021-12-21

**Authors:** Menghuai Sun, Qian Lin, Chunyang Wang, Jiao Xing, Kunlong Yan, Zhifeng Liu, Yu Jin, Carol J. Cardona, Zheng Xing

**Affiliations:** ^1^Medical School and Jiangsu Provincial Key Laboratory of Medicine, Nanjing University, Nanjing, China; ^2^Nanjing Children’s Hospital, Nanjing Medical University, Nanjing, China; ^3^Department of Gastroenterology, Beijing Children’s Hospital, Capital Medical, University, National Center for Children’s Health, China; ^4^Clinical Medical College, Xi’an Medical University, Xi’an, China; ^5^Department of Veterinary Biomedical Sciences, College of Veterinary Medicine, University of Minnesota at Twin Cities, Saint Paul, MN, United States

**Keywords:** Enterovirus (EV) A71, KPNA1, IFN-α, karyopherin-α 1, 2B

## Abstract

Enterovirus A71 (EV-A71) is a major pathogen that causes the hand, foot, and mouth disease, which could be fatal with neurological complications in children. The underlying mechanism for the severe pathogenicity remains obscure, but impaired or aberrant innate immunity is considered to play a key role in viral pathogenesis. We reported previously that EV-A71 suppressed type I interferon (IFN) responses by inducing degradation of karyopherin-α1 (KPNA1), a component of the p-STAT1/2 complex. In this report, we showed that 2B, a non-structural protein of EV-A71, was critical to the suppression of the IFN-α-induced type I response in infected cells. Among viral proteins, 2B was the only one that was involved in the degradation of KPNA1, which impeded the formation of the p-STAT1/2/KPNA1 complex and blocked the translocation of p-STAT1/2 into the nucleus upon IFN-α stimulation. Degradation of KPNA1 induced by 2B can be inhibited in the cells pre-treated with Z-DEVD-FMK, a caspase-3 inhibitor, or siRNA targeting caspase-3, indicating that 2B-induced degradation of KPNA1 was caspase-3 dependent. The mechanism by which 2B functioned in the dysregulation of the IFN signaling was analyzed and a putative hydrophilic domain (H1) in the N-terminus of 2B was characterized to be critical for the release of cytochrome c into the cytosol for the activation of pro-caspase-3. We generated an EV-A71 infectious clone (rD1), which was deficient of the H1 domain. In rD1-infected cells, degradation of KPNA1 was relieved and the infected cells were more sensitive to IFN-α, leading to decreased viral replication, in comparison to the cells infected with the virus carrying a full length 2B. Our findings demonstrate that EV-A71 2B protein plays an important role in dysregulating JAK-STAT signaling through its involvement in promoting caspase-3 dependent degradation of KPNA1, which represents a novel strategy employed by EV-A71 to evade host antiviral innate immunity.

## Introduction

Enterovirus 71 (EV-A71) is a non-enveloped, positive-sense, and single-stranded RNA virus, which belongs to the human enterovirus species A of the genus *Enterovirus* within the *Picornaviridae* family (Brown and Pallansch, 1995). EV-A71 is a major causative agent of the hand, foot and mouth disease (HFMD) in young children and infants. Acute infection with EV-A71 can cause severe neurological complications or even fatal diseases (McMinn, 2002). Since its initial isolation and identification in 1969 (Schmidt et al., 1974), EV-A71 has caused large-scale epidemics in the world, especially in the Asia-Pacific region (Chan et al., 2000; McMinn et al., 2001; Xing et al., 2014), and posed a serious health threat to children.

Type I IFNs, IFN-α and IFN-β, are critical to innate immunity against viral infections and play an important role in suppressing viral replication. IFNs function through hundreds of interferon-stimulated genes (ISGs) (Platanias, 2005; Gonzalez-Navajas et al., 2012). As the first line of defense against viral infection, type I interferon response is initiated after IFNs bind to heterodimeric receptors, consisting of interferon-α receptors 1 (IFNAR1) and 2 (IFNAR2) on the cell surface, leading to the activation of the tyrosine kinase 2 (Tyk2) and Janus kinase 1 (Jak1). Both signal transducer and activator of transcription 1 (STAT1) and STAT2 are recruited to the receptor-bound Jaks and phosphorylated at tyrosine. The phosphorylated STAT1/2 form heterodimers which interact with interferon regulatory factor 9 (IRF9) and subsequently form a heterotrimeric complex, also known as interferon-stimulated gene factor 3 (ISGF3). Translocation of ISGF3 into the nucleus and subsequent binding to the IFN-stimulated response elements (ISREs) lead to transcription and expression of ISGs.

IFNs, as powerful therapeutic antivirals, have been applied to treatment of many viral infections clinically (Platanias, 2005). Unexpectedly, however, IFNs have little effect clinically on patients infected with EV-A71 at regular concentrations (Lu et al., 2012). Accumulated evidence indicates that EV-A71 infection can circumvent the IFN response by either suppressing IFN induction or blocking induction of ISGs (Lei et al., 2010; Wang B. et al., 2013; Zhang et al., 2014). The mechanisms underlying EV-A71 resistance to type I IFNs are not thoroughly understood and several theories have been proposed. It was suggested that EV-A71 inhibited the type I IFNs signaling by down-regulating expression of IFNAR1 (Lu et al., 2012). One study reported that EV-A71 suppressed the IFN response through down-regulating JAK1 while the level of IFNAR1 was not changed significantly in cells infected with EV-A71 (Liu et al., 2014).

Karyopherins (KPNAs), or importins, are a set of cytoplasmic proteins that recognize nuclear localization signals (NLSs) and are capable of docking NLS-containing proteins to the nuclear pore complex (Quensel et al., 2004; Nitahara-Kasahara et al., 2007; Yasuhara et al., 2007; Hall et al., 2011). As KPNAs are essential for the nuclear transport of p-STAT1/2 and translocation of the ISGF complex, they have been frequently targeted by various viruses to antagonize the JAK/STAT pathway and facilitate their replication. VP24 of Ebola virus was reported to bind KPNA1 and KPNA5 and disrupt the interaction between p-STAT1 and KPNA1, thereby preventing p-STAT1 from nuclear translocation and antagonizing IFN signaling (Reid et al., 2006; Ding et al., 2017). Interestingly, different Ebola viruses exhibit distinct VP24-KPNA1 binding affinities, which may be correlated to their differences in virulence (Schwarz et al., 2017). The foot-and-mouth disease virus (FMDV) 3Cpro induces degradation of KPNA1 in a proteasome- and caspase-independent manner while nsp1β of porcine reproductive and respiratory syndrome virus (PRRSV) promotes KPNA1 degradation through an ubiquitin-proteasome pathway (Wang R. et al., 2013; Du et al., 2014).

We have previously shown that KPNA1 was degraded in EV-A71-infected cells and caspase-3 activity was somehow involved in the degradation of KPNA1, resulting in the suppression of the ISG transcription. However, the exact mechanism about how EV-A71 is resistant to IFN has not been fully elucidated. In this study we report that 2B, a viral non-structural protein of EV-A71, was involved in the degradation of KPNA1. The induced degradation of KPNA1 could be prevented when caspase-3 activity was inhibited, indicating that 2B may promote KPNA1 degradation in a caspase-3 dependent manner. We showed that a putative N-terminal hydrophilic domain (H1) of 2B was essential for the activation of pro-caspase-3 and degradation of KPNA1, which led to the suppression of JAK-STAT signaling. Our findings provide further insights into our understanding of the mechanisms employed by EV-A71 to counteract the type I IFN-mediated antiviral responses.

## Materials and Methods

### Cells and Virus

HeLa, HT-29, RD, and Vero cells were purchased from the Cell Bank of the Chinese Academy of Sciences (Shanghai, China). The cells were maintained in Dulbecco’s modified Eagle’s medium (DMEM) with high glucose (Gibco, Carlsbad, CA, United States), supplemented with 10% FBS (Gibco), and were cultured at 37°C in a humidified atmosphere with 5% CO_2_. EV-A71 Fuyang 0805 strain (FY EV-A71, NCBI accession number FJ439769), which belongs to the C4a cluster of the Enterovirus C4 subgenotype as verified by sequencing analysis of the VP1 region, was provided by Dr. Bin Wu, Jiangsu Provincial Centers of Disease Control, and propagated on Vero cells. Recombinant rFY and rD1 EV-A71 viruses were generated through a reverse genetics system based on the genome of the Fugyang 0805 strain. All viruses were used to inoculate the cells at a multiplicity of infection (MOI) of 1 unless stated otherwise. Virus titers were presented as the median (50%) tissue culture infectious doses (TCID_50_) determined in Vero cells as described previously (Kärber, 1931).

### Reagents and Plasmids

For IFN stimulation, recombinant human IFN-α1 (#8927, Cell Signaling Technology or CST, Boston, MA, United States) and recombinant human IFN-β1b (P5660, Beyotime, Shanghai, China) was added to the cultured cells at a final concentration of 25 ng/μL unless stated differently. Caspase inhibitors, including Z-DEVD-FMK (caspase-3), Z-VEID-FMK (caspase-6), AC-LEVD-CHO (caspase-4), Z-IETD-FMK (caspase-8), and Q-VD-OPH, a broad-spectrum caspase inhibitor, were purchased from CalBiochem (San Diego, CA, United States). Proteasome inhibitors, 3-MA and MG132, were purchased from Sigma-Aldrich (St. Louis, MO, United States). Cisplatin, an apoptosis inducer, was purchased from Beyotime. Pro-caspase activating compound 1 (PAC1), a caspase-3 agonist, was ordered from Tocris Bioscience (Bristol, United Kingdom).

Antibodies specific for MX1 (#37849, CST), OAS1 (#14498, CST), hemagglutinin (HA) (#3724, CST), hemagglutinin (HA) (ab18181, Abcam, Cambridge, United Kingdom), β-Tubulin (BS1482, Bioworld Technology, St Louis Park, MN, United States), phosphorylated STAT1 at tyrosine-701 (p-STAT1) (#9167, CST), phosphorylated STAT2 at tyrosine-690 (p-STAT2) (#88410, CST), Histone H3 (#4499, CST), EV-A71 VP1 (ab169442, Abcam), green fluorescence protein (GFP; #2956, CST), FLAG (ab125243, Abcam), PARP (#9532, CST), cleaved-PARP (#5625, CST), pro-caspase-9 (#9504, CST), cleaved-caspase-9 (#7237, CST), pro-caspase-8 (#4790, CST), cleaved-caspase-8 (#9496, CST), pro-caspase-3 (#14220, CST), cleaved-caspase-3 (#9664, CST) cytochrome c (#4280, CST), COX IV (#4850, CST), HRP-linked anti-rabbit IgG (#7074, CST), and HRP-linked anti-mouse IgG (#7076, CST), were used in western blot analyses.

A keyhole limpet hemocyanin conjugated peptide (GVSDYIKGLGDAFGC) was designed according to the immunogenicity prediction of the EV-A71 2B protein and synthesized by GenScript (Nanjing, China). New Zealand rabbits were inoculated with this peptide immunogen to produce polyclonal antibodies against EV-A71 2B, which were used to perform western blot analyses or immunofluorescence assays (IFA) (1:500-1:1,000 dilution).

Antibodies specific for HA tag (ab18181, Abcam), p-STAT1 antibody (sc-136229, Santa Cruz Biotechnology, Santa Cruz, CA, United States), anti-KPNA1 antibody (sc-101292, Santa Cruz Biotechnology), FLAG tag (ab125243, Abcam), pro-caspase-3 (ab32150, Abcam) and normal rabbit/mouse IgG (A7016/A7028, Beyotime) were used for immunoprecipitation. Antibodies specific for p-STAT1 (#9167, CST), p-STAT1 (sc-8394, Santa Cruz Biotechnology), HA tag (ab18181, Abcam), pro-caspase-3 (ab32150, Abcam), EV-A71 VP1 (ab36367, Abcam), goat anti-rabbit IgG (H + L) (FITC conjugate; A0562, Beyotime), goat anti-mouse IgG (H + L) (Cy3 conjugate; A0521, Beyotime), goat anti-mouse IgG (H + L) (FITC conjugate; A0568, Beyotime), goat anti-rabbit IgG (H + L) (Cy3 conjugate; A0516, Beyotime) and MitoTracker™ Deep Red FM (M22426, Thermo Fisher Scientific, Waltham, MA, United States) were used for immunofluorescence assay.

The sequences for small inhibitory RNA (siRNA) molecules targeting human pro-caspase-3 were 5′- AGUGAAGCAAAUCAGAAACTT-3′ and 5′-GUUUCUGAUUUGCUUCACUTT-3′, and for a scramble siRNA as control were 5′-UUCUCCGAACGUGUCACGUTT-3′ and 5′-ACGUGACACGUUCGGAGAATT-3′. siRNAs were synthesized by Genepharma Inc., (Shanghai, China). Plasmids, pcDNA3.1-3xFlag-KPNA1, pcDNA3.1-3xFlag-KPNA2, pcDNA3.1-3xFlag-KPNA3, pcDNA3.1-3xFlag-KPNA4, and pcDNA3.1-EFGP-STAT1 were obtained from YouBio (Changsha, China). To construct plasmids expressing VP1, VP2, VP3, VP4, 2A, 2B, 2C, 3A, 3B, 3C, or 3D proteins of EV-A71, corresponding cDNA fragments of the EV-A71 Fuyang 0805 strain were cloned into *Bam*HI and *Sal*I sites of pRK5-HA, resulting in various constructs expressing the viral proteins fused to the HA tag. Several plasmids expressing truncated 2B fragments were constructed with Mut Express II Fast Mutagenesis Kit V2 (Vazyme, Nanjing, China). All of the plasmids were confirmed by restriction enzyme digestion and DNA sequencing.

### Western Blot Analysis

Cell lysates were prepared from infected or uninfected cells at indicated time points post infection (p.i.) with the Radio Immunoprecipitation Assay (RIPA) lysis buffer (Beyotime) supplemented with proteases and phosphatase inhibitors (Roche, Basel, Switzerland). Protein samples were electrophoresed by sodium dodecyl sulfate-polyacrylamide gel electrophoresis (SDS-PAGE). The separated proteins were transferred onto a polyvinylidene difluoride (PVDF) membrane (Bio-Rad, Hercules, CA, United States) and probed with primary antibodies, which were further bound to HRP-conjugated secondary antibodies after washes. Finally, the PVDF membrane was immersed by Immun-Star™ HRP Substrate (Bio-Rad, Hercules, CA, United States) and images were captured using a ChemiDoc™ XRS + imaging system (Bio-Rad). Digital signal acquisition and analysis were conducted using the Quantity One program, version 4.6 (Bio-Rad).

### Immunoprecipitation

Cells were lysed with a lysis buffer (50 mM Tris, pH 7.4, 150 mM NaCl, 0.2 mM EDTA, 2 mM EGTA, 0.5% Igepal CA-630, 10% glycerol, and 1 mM sodium vanadate) supplemented with a protease inhibitor cocktail (Roche). The cell lysates, after quantification, were used for incubation with either pre-immune serum or specific antibodies at 4°C overnight, followed by incubation with protein A/G agarose (EMD Millipore Corporation, Temecula, CA, United States). The agarose beads were thoroughly washed four times and the immuneprecipitates were subjected to western blot analyses with specific antibodies.

### RNA Isolation and Real-Time PCR

Total RNA was prepared from cells with TRIzol reagent (Invitrogen, Grand Island, NY, United States) in accordance with the manufacturer’s instructions. The quantity and purity of total RNA were measured by a Nanodrop 2000 spectrophotometer (Thermo Fisher Scientific). The RNA was reverse transcribed for cDNA using SuperScript™ IV VILO™ Master Mix (Invitrogen). Real-time PCR was carried out using primers specific for human MX1, MX2, OAS1, IFI27, ISG54, ISG56, or KPNA1. Reactions consisted of 10 μL 2 × Power SYBR Green PCR Master Mix (ABI, Foster City, CA, United States) and primers in 20 μL. Quantitative PCR was performed by QuantStudio™ 5 Real-Time PCR System (ABI). Data were calculated as fold change (2^–ΔΔCt^), which was the relative level of corresponding gene transcripts normalized to an internal control, human glyceraldehyde-3-phosphate dehydrogenase (GAPDH). For examining EV-A71 replication, an RNA fragment corresponding to nucleotides 2462-2635 (VP1 region) of the Fuyang 0805 strain was adjusted to a concentration gradient (1 × 10^1^ to 1 × 10^8^ copies/μL) used as standards to calculate the copy number of viral RNA in the quantitative PCR. The primers used were EV-A71-F (5′-AGATAGGGTGGCAGATGTAATTGAAAG-3′) and EV-A71-R (5′-TAGCATTTGATGATGCTCCAAT-3′).

### Immunofluorescence Assay and Confocal Fluorescence Microscopy

Infected or normal cells, seeded onto coverslips, were washed twice with phosphate buffered saline (PBS) and fixed with 4% paraformaldehyde in PBS. The cells were premeabilized with 0.1% Triton X-100 in PBS and blocked with 5% bovine serum albumin (BSA) at 37°C for 1 h before incubation with primary antibodies in PBS containing 1% BSA overnight at 4°C, followed by three washes in PBS. The cells were subsequently incubated with secondary antibodies at 37°C for 1 hr. The cells were washed and incubated with 5 μg/mL of 4′, 6′-diamidino-2-phenylindole (DAPI) diluted in PBS for 10 min. After three washes with PBST, the cells were covered with one droplet of Prolong™ Gold Antifade Mountant (Molecular Probes, Eugene, OR, United States) and observed under a FV3000 Confocal Laser Scanning Microscope (Olympus, Tokyo, Japan).

### Caspase Activity Assay

Cytosolic lysates of cells were prepared and normalized by a Bradford assay according to the instructions of the caspase-3 and -9 activity assay kits (C1116 and C1157, Beyotime). Ac-DEVD-pNA and Ac-LEHD-pNA were used as the substrates for caspase-3 and -9, respectively. Absorbance at OD_405_ (A_405 nm_) was determined in a Multiskan FC (Thermo Fisher Scientific) and the relative caspase activity was determined as a percentage of the value from a positive control.

### Cell Viability Test

Cell viability was measured by a cell counting kit-8 (CCK-8, Beyotime). Briefly, HeLa cells were seeded onto 96-well plates at a density of 2 × 10^3^ cells/well. The cells were treated with Cisplatin (30 μM) or PAC1 (50 μM), or transfected with plasmids expressing HA-tagged 2B, for 36 h at 37°C. 10 μl of CCK-8 solution was added to each well. After incubating CCK-8 in the cell culture at 37°C for 30 min, the optical density (OD) of the supernatant in the culture was measured at 450 nm with a Multiskan FC (Thermo Fisher Scientific). Survival rates of cells were calculated and expressed as the ratio of the absorbance at OD450 (A_450_) of the infected cells to that of the untreated cells. The assay was performed in triplicates for each time points.

### EV-A71 Virus Produced From EV-A71 Infectious Clones

The complete genomic cDNA of EV-A71 Fuyang 0805 strain was chemically synthesized and cloned into pcDNA3.1(+) between Hind III and Sal I (GenScript) for producing a rFY EV-A71 infectious clone. To generate mutant viruses deficient of a viral 2B H1 domain, a truncated EV-A71 infectious clone, rD1, was constructed by Mut Express II Fast Mutagenesis Kit V2 (Vazyme, Nanjing, China) using the rFY EV-A71 cDNA as the template. RNA transcripts were synthesized *in vitro* from linearized cDNA using MEGAscript^®^ T7 Kit (Thermo Fisher Scientific). The RNA transcripts were transfected into Vero cells using Lipofectamine^®^ 3000 (Thermo Fisher Scientific). On day 7 post-transfection, supernatants containing the virus from the transfected cells were collected and infectious viruses titrated. The recombinant virus was aliquoted and stored at −80°C for use.

### Statistical Analysis

The two-tailed unpaired Student’s *t*-test was used to analyze the data. The data shown are the means ± standard deviations (SD) of repeated experiments. The difference was considered statistically significant when *p* value was less than 0.05.

## Results

### EV-A71 2B Inhibited ISG Induction and p-STAT1/2 Translocation Stimulated by IFN-α

**FIGURE 1:**
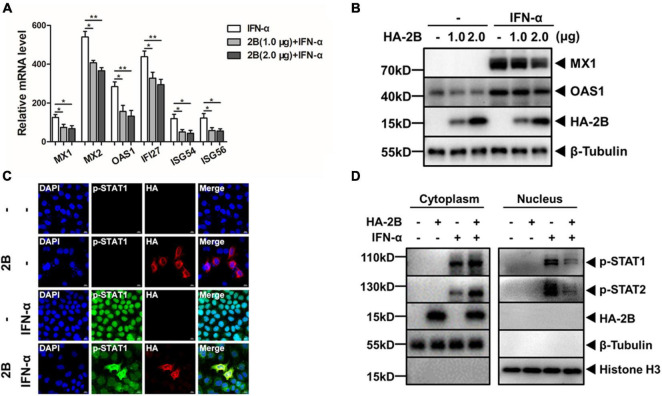
EV-A71 2B inhibited type I interferon responses. HeLa cells were transfected with plasmids expressing HA-tagged 2B, followed by treatment with or without IFN-α (25 ng/μL). **(A)** EV-A71 2B inhibited the induction of ISGs induced by IFN-α. Total RNA was prepared from the cells for real-time RT-PCR to measure ISG mRNA transcripts. Values represent mean ± SD from triplicates of independent experiments.*P<0.05; **P<0.01. **(B)** EV-A71 2B inhibited the induction of MX1 and OAS1. The cell lysates were prepared for western blot analyses with antibodies for MX1 and OAS1. **(C)** EV-A71 2B prevented p-STAT1 from translocation into the nucleus upon IFN-α stimulation. The cells were fixed and premeabilized for subsequent staining with antibodies for HA tag and p-STAT1, which were subjected to confocal microscopy. Magnification, × 630. **(D)** Translocation of p-STAT1 and p-STAT2 was blocked by 2B. The cytosolic and nuclear fractions were prepared from the cell lysates, which were further subjected to western blot analyses with antibodies specific for p-STAT1 and p-STAT2.

Our previous study showed that IFN-β failed to inhibit EV-A71 replication in Vero cells and EV-A71 inhibited the induction of ISGs stimulated by IFN-β in HeLa cells (Wang C. et al., 2017). To further examine the mechanism about how EV-A71 plays the pivotal role in suppressing type I IFN signaling, we attempted to decipher the function of individual EV-A71 proteins on the induction of ISGs by IFN-α treatment in HeLa cells. We started with 2B, which is one of the seven non-structural proteins encoded by EV-A71 genome. Cells were transfected with a plasmid expressing HA-tagged viral 2B protein, followed by treatment with IFN-α. Transcription of IFN responsive genes was examined in total RNA prepared from the transfected cells by real-time RT-PCR. As shown in Figure 1A, the mRNA transcript levels of MX1, MX2, OAS1, IFI27, ISG54, and ISG56 increased up to 125, 540, 284, 438, 119, and 122-fold, respectively, in IFN-α-treated cells compared with those in untreated cells. However, in IFN-α-treated cells in the presence of viral 2B, the induction of these ISGs was significantly lower in comparison to the cells absent of viral 2B. Cell lysates were prepared from the cells expressing 2B, followed by simulation of IFN-α, for western blot analyses. The induction of MX1 and OAS1 by IFN-α stimulation was suppressed in the presence of 2B (Figure 1B). These data combined indicate that the induction of ISGs by IFN-α was inhibited by viral 2B.

Previously we showed that EV-A71 infection did not alter the expression of IFNAR1 or JAK1, neither did it induce the degradation of STAT1 or STAT2 (Wang C. et al., 2017). Instead we demonstrated that the translocation of p-STAT1/p-STAT2 from the cytosol to the nucleus was blocked upon the type I IFN stimulation in EV-A71-infected cells. To examine whether 2B protein was involved in preventing the translocation of p-STAT1/2 in JAK/STAT signaling, we stimulated HeLa cells, in the presence or absence of viral 2B, with IFN-α and examined the subcellular localization of p-STATs under a laser confocal microscope. Our data showed that p-STAT1 was present mainly in the nucleus upon IFN-α stimulation. However, in the presence of 2B, p-STAT1 remained predominantly in the cytoplasm in the cells stimulated with IFN-α (Figure 1C). We fractionated the cell lysates, prepared from the transfected cells stimulated with or without IFN-α, and collected the nuclear and cytoplasmic fractions, respectively, for western blot analyses. As shown in Figure 1D, p-STAT1/p-STAT2 were mainly detected in the nuclear fraction in the cells absent of 2B upon IFN-α stimulation. In the presence of 2B, however, p-STAT1 and p-STAT2 were mostly retained in the cytoplasmic fraction. These results demonstrate that viral 2B may block the translocation of p-STAT1/p-STAT2 from the cytosol to the nucleus in IFN-α stimulated cells.

### EV-A71 2B Reduced KPNA1 Levels and p-STAT1/KPNA1 Complex

**FIGURE 2:**
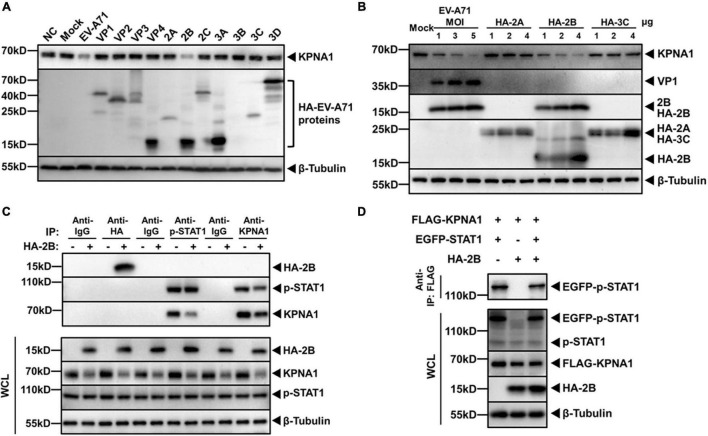
EV-A71 2B reduced levels of KPNA1 and p-STAT1/KPNA1 complex. **(A)** Reduction of KPNA1 levels by EV-A71 viral proteins. HeLa cells were infected with EV-A71 at an MOI of 1 or transfected with plasmids (2.0 μg) expressing HA-tagged viral proteins. Cell lysates were prepared for western blot analyses with antibodies for HA tag or KPNA1. **(B)** Reduction of KPNA1 levels by viral 2A, 2B, and 3C. HeLa cells were infected with EV-A71 or transfected with plasmids expressing HA-tagged 2A, 2B, or 3C. Cell lysates were prepared for western blot analyses with antibodies for HA tag and KPNA1. **(C)** Reduction in levels of the p-STAT1 and KPNA1 complex in IFN-α-stimulated cells by 2B. HeLa cells were transfected with plasmids expressing HA-tagged 2B (2.0 μg). At 36 h post transfection, the cells were treated with IFN-α (25 ng/μL) for 2 h. Cell lysates were prepared for co-immunoprecipitation (Co-IP) with antibodies for HA, p-STAT1, or KPNA1. Normal isotype IgG was used as negative control. Subsequent western blot analyses were performed with antibodies for HA, p-STAT1, or KPNA1. The levels of inputs in the whole cell lysates (WCL) were also shown together with β-Tubulin. **(D)** EV-A71 2B reduced the levels of the exogenous p-STAT1 and KPNA1 complex. HeLa cells were transfected with plasmids expressing FLAG-KPNA1 (2.0 μg), EGFP-STAT1 (2.0 μg), and HA-2B (2.0 μg). At 36 h post transfection, the cells were treated with IFN-α (25 ng/μL) for 2 h. Cell lysates were prepared for Co-IP with an anti-FLAG antibody and subsequent western blot analyses with antibodies for EGFP tag. The levels of inputs in WCL were also shown with antibodies specific for FLAG, EGFP, or HA tags.

We previously observed that the levels of KPNA1 decreased in RD, Vero or HT-29 cells infected by EV-A71 (Wang C. et al., 2017). KPNA1 plays a role in p-STAT1 nuclear translocation by recognizing and targeting p-STAT1 and other substrates destined for nuclear import. In this study we aimed to determine whether some viral protein of EV-A71 may directly be involved in targeting KPNA1. HeLa cells were infected with EV-A71 and cell lysates were prepared for western blot analyses, which showed a decreased level of KPNA1. We transfected HeLa cells with plasmids expressing VP1, VP2, VP3, VP4, 2A, 2B, 2C, 3A, 3B, 3C, or 3D and cell lysates were analyzed likewise. As shown in Figure 2A, among all cell lysates, a significantly lower level of KPNA1 was observed only from the cells expressing viral 2B, which was comparable to the decreased level in EV-A71-infected cells.

The cells that expressed viral proteins 2A, 2B, or 2C by transfection, and the cells that were infected with EV-A71 were further analyzed. Viral non-structural proteins 2A and 3C possess proteinase activity, which can cleave viral proteins or host factors responsible for the IFN response (Hung et al., 2011; Lei et al., 2011, 2013; Wang B. et al., 2013; Feng et al., 2014). It appeared that 2A and 3C had no apparent effect on the levels of KPNA1. KPNA1 decreased in levels only in the cells expressing 2B or infected with EV-A71 (Figure 2B).

When phosphorylated, p-STAT1 interacts with KPNA1, resulting in a formation of heterotrimers with IRF9 for nuclear translocation. We examined whether viral 2B could have any effect on the interaction of p-STAT1 and KPNA1. HeLa cells were transfected with plasmids expressing 2B, followed by IFN-α stimulation. Cell lysates were prepared for Co-IP with antibodies for p-STAT1 or KPNA1 and the immunoprecipitates were subjected to western blot analysis. As shown in Figures 2C, 2B did not interact with either p-STAT1 or KPNA1, while p-STAT1 was bound to KPNA1 in IFN-α-stimulated cells. On the other hand, we did find that the levels of p-STAT1/KPNA1 complex declined when viral 2B was present in the cells (Figure 2C). Similar results were also observed with the complex of p-STAT1 and KPNA1, which were exogenously expressed in the cells (Figure 2D). Taken together with the results shown earlier, our data indicate that EV-A71 2B caused the reduction of the KPNA1 levels, leading to decreased formation of the p-STAT1/KPNA1 complex in the cells stimulated by IFN-α.

### EV-A71 2B Targeted KPNA1 Specifically

**FIGURE 3:**
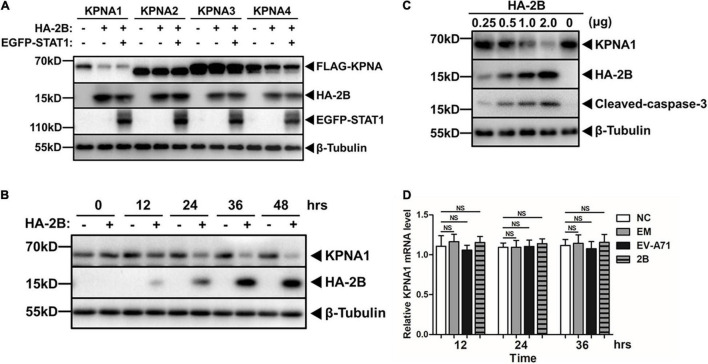
Reduction of KPNA1 was specific among KPNA family members by 2B. **(A)** Viral 2B had minimal effect on KPNA2, KPNA3, and KPNA4 levels. HeLa cells were co-transfected with plasmids expressing HA-tagged 2B, EGFP-tagged STAT1 and FLAG-tagged KPNA1, FKPNA2, KPNA3, or KPNA4 (2.0 μg each) for 36 h, followed by treatment of IFN-α (25 ng/μL) for another 2 h. Cell lysates were prepared for western blot analyses with antibodies for FLAG, HA, EGFP, or β-Tubulin. **(B,C)** Dynamics in the reduction of KPNA1 levels by 2B. HeLa cells were transfected with plasmids expressing HA-tagged 2B and cell lysates were prepared at various time points post transfection **(B)**. HeLa cells were transfected with varying amount of plasmids and cell lysates were prepared 36 h post transfection **(C)**. The cell lysates were analyzed by western blot analyses with antibodies for KPNA1, HA, or β-Tubulin. **(D)** Transcription of KPNA1 was not affected by 2B. HeLa cells were transfected with plasmids expressing HA-tagged 2B or infected with EV-A71 at an MOI of 1. Total RNA were prepared from the cells for real-time RT-PCR to measure mRNA transcripts of KPNA1 with specific primers. Values represent mean ± SD from triplicates of independent experiments. NS, no significance; NC, normal control; EM, empty vector control.

We were interested in understanding whether the degradation of KPNA1 by EV-A71 2B was specific for only KPNA1, or a broad effect on all KPNA family proteins. We collected the cell lysates from HeLa cells co-transfected with plasmids expressing tagged KPNA1, KPNA2, KPNA3, or KPNA4 as well as viral 2B and STAT1. Our data showed that viral 2B only targeted KPNA1 while it had no or marginal effect on KPNA2, KPNA3, or KPNA4. In addition, expression of STAT1 seemed to have no effect on the reduction of KPNA1 by viral 2B (Figure 3A).

To further confirm the reduction of KPNA1 by viral 2B, we prepared cell lysates from the cells expressing 2B at various time points post transfection for western blot analyses. As shown in Figure 3B, while the expression of viral 2B increased over time, the levels of KPNA1 decreased gradually. When the expression of 2B increased in the cells transfected with increasing amounts of plasmids encoding 2B, the levels of KPNA1 decreased (Figure 3C), indicating that KPNA1 was reduced by viral 2B in a time- and dose-dependent manner.

We tried to understand at which phase, transcriptional or post-transcriptional, the expression of KPNA1 was affected in the cells expressing viral 2B. Total RNA was prepared at various time points from the cells, either infected with EV-A71 or transfected with plasmids expressing 2B, for real-time RT-PCR to determine the levels of KPNA1 mRNA transcripts. As shown in Figure 3D, no significant changes of the KPNA1 mRNA levels were observed in EV-A71-infected cells or the cells exogenously expressing 2B as compared to the control cells, indicating that the reduction of KPNA1 by viral 2B may occur at the post-transcriptional level.

### Caspase-3 Was Involved in KPNA1 Reduction

**FIGURE 4:**
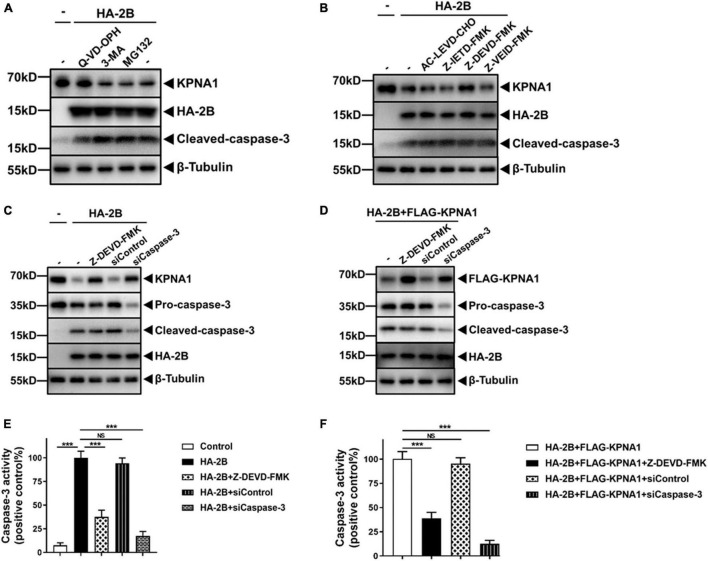
Caspase-3 was involved in KPNA1 degradation induced by 2B. **(A)** Degradation of KPNA1 was prevented from degradation by a caspase inhibitor. HeLa cells were pre-treated with a broad-spectrum caspase inhibitor, Q-VD-OPH (20 nM), an autophagy inhibitor, 3-MA (10 mM), or a proteasome inhibitor, MG132 (5 μM), for 12 h, followed by transfection with plasmids expressing HA-tagged 2B (2.0 μg) for another 36 h. The whole cell lysates were prepared for western blot analyses with antibodies specific for KPNA1, HA tag, and cleaved-caspase-3. **(B)** A caspase-3 specific inhibitor prevented KPNA1 from degradation. HeLa cells were pre-treated with inhibitors specific for caspase-4 (AC-LEVD-CHO, 30 μM), caspase-8 (Z-IETD-FMK, 30 μM), caspase-3 (Z-DEVD-FMK, 100 μM), and caspase-6 (Z-VEID-FMK, 50 μM) for 12 h prior to transfection with HA-2B plasmid (2.0 μg). The whole cell lysates were prepared 36 h post transfection for western blot analyses with antibodies for KPNA1, HA tag, and cleaved caspase-3. **(C,D)** Degradation of KPNA1 was caspase-3 dependent. HeLa cells were transfected with siRNA specific for caspase-3, or a scramble RNA as control (siRNA final, 50 nM), or pre-treated with an inhibitor specific for caspase-3 (Z-DEVD-FMK, 100 μM), for 24 h, followed by transfection of plasmids expressing HA-tagged 2B alone **(C)** or by co-transfection of plasmids expressing HA-tagged 2B and FLAG-tagged KPNA1 **(D)** for another 36 h. Cell lysates were prepared for western blot analyses with antibodies for KPNA1, HA tag, pro-caspase-3, or cleaved-caspase-3. Degradation of endogenous and exogenous KPNA1 was shown in panels **(C,D)**, respectively. **(E,F)** Relative caspase-3 enzymatic activities were detected in cells expressing 2B. Cell lysates were prepared from the cells with different treatments for the caspase-3 activity assay. The cells were either transfected with plasmids to expressing HA-2B alone **(E)** or co-transfected with plasmids to expressing HA-2B and KPNA1 **(F)**. The values represented mean ± SD from triplicates of independent experiments. Cells transfected with HA-2B **(E)** and HA-2B + FLAG-KPNA1 **(F)** were used as positive controls. Results of *t* tests were shown as transverse lines as indicated. NS, no significance; ***P < 0.001.

We reasoned that viral 2B may reduce the levels of KPNA1 post-transcriptionally through promoting its degradation at protein levels. Cellular proteins are commonly degraded by ubiquitin-proteasome-dependent degradation, the autophagy-lysosomal pathway, or caspase-mediated proteolysis (Rubinsztein, 2006). To assess which pathway may be involved in the 2B-mediated degradation of KPNA1, we pre-treated HeLa cells with a proteasome inhibitor, MG132 (5 μM), an autophagy inhibitor, 3-methyladenine (3-MA, 10 mM), or a broad-spectrum caspase inhibitor, quinoline-Val-Asp-difluorophenoxymethylketone (Q-VD-OPH, 20 nM) for 12 h, followed by transfection of the plasmid expressing viral 2B. Cell lysates were harvested at 36 h post transfection for western blot analyses. As shown in Figure 4A, the degradation of KPNA1 by 2B was significantly blocked in the cells pre-treated with Q-VD-OPH, whereas the levels of KPNA1 remained unchanged in the cells pre-treated with either MG132 or 3-MA in the presence of viral 2B, suggesting that activities of caspase were involved in the degradation of KPNA1 induced by viral 2B.

Previously we reported that the degradation of KPNA1 induced by EV-A71 infection was mediated by activated caspase-3 but had no clue about how caspase-3 was activated (Wang C. et al., 2017). To understand whether viral 2B was involved in the activation of caspase-3, we first examined whether caspase-3 or other caspases may be responsible for the degradation of KPNA1 triggered by viral 2B. HeLa cells were pre-treated with inhibitors for caspase-3 (Z-DEVD-FMK, 100 μM), caspase-4 (AC-LEVD-CHO, 30 μM), caspase-6 (Z-VEID-FMK, 50 μM), or caspase-8 (Z-IETD-FMK, 30 μM), followed by transfection with plasmids expressing viral 2B. Cell lysates were prepared at 36 h post transfection for western blot analyses. The data showed that the degradation of KPNA1, induced by 2B, was significantly suppressed only in the cells pre-treated with Z-DEVD-FMK, the inhibitor of caspase-3, whereas the inhibitors for caspase-4, -6, or -8 had no obvious effect on the levels of KPNA1 (Figure 4B).

To confirm the role of caspase-3 in the process of KPNA1 degradation by 2B, HeLa cells were pre-treated with Z-DEVD-FMK to inhibit enzymatic activity of caspase-3 or transfected with siRNA, specific for targeting pro-caspase-3 to knock down pro-caspase-3 mRNA, followed by transfection of plasmids expressing 2B. The results showed that both pre-treatment with Z-DEVD-FMK and siRNA knockdown of pro-caspase-3 protected the endogenous KPNA1 from degradation (Figure 4C). This result was reproducible on exogenously expressed KPNA1 in the cells which were either pre-treated with Z-DEVD-FMK or knocked down with pro-caspase-3 siRNA (Figure 4D).

It was worth mentioning that 2B expression induced cleavage of pro-caspase-3, which was not affected by multiple inhibitors abovementioned. However, the caspase-3 activity was sharply curtailed in the cells treated with Z-DEVD-FMK as shown in Figures 4E,F, further indicating that the KPNA1 degradation by viral 2B was dependent on proteinase activity of caspase-3. We noted that siCaspase-3 treatment led to a much weaker caspase-3 activity (Figures 4E,F), which was apparently attributed to decreased levels of pro-caspase-3 mRNA and protein, different from the treatment with Z-DEVD-FMK.

### 2B Protein Induced KPNA1 Degradation in Various Cell Types and Intrinsic Apoptosis

**FIGURE 5:**
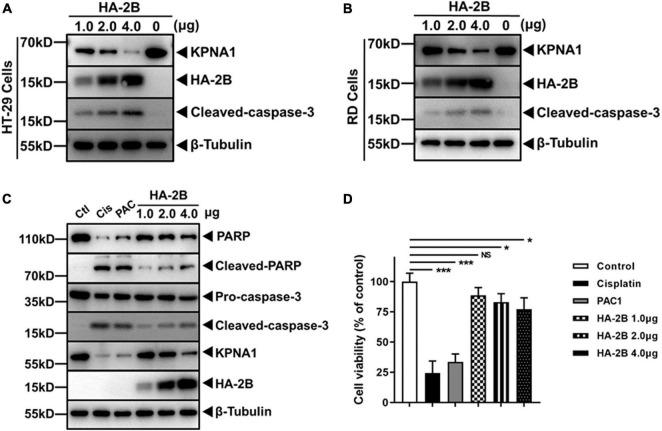
Viral 2B induced KPNA1 degradation in various cell types and intrinsic apoptosis. **(A,B)** Degradation of KPNA1 and cleaved-caspase-3 were detected in HT-29 **(A)** and RD **(B)** cells. Cells transfected with plasmids expressing HA-tagged 2B and cell lysates were prepared 36 h post transfection with varying amounts of plasmids for western blot analyses with antibodies for KPNA1, HA, cleaved-caspase-3, or β-Tubulin. **(C)** 2B induced PARP cleavage as well as KPNA1 degradation. HeLa cells were treated with Cisplatin (30 μM) or PAC1 (50 μM), or transfected with different amounts of plasmids expressing HA-tagged 2B for 36 h. Whole cell lysates were collected for western blot analyses with specific antibodies. Ctl, untreated group; Cis, Cisplatin; PAC, PAC1. **(D)** Cell viabilities with 2B expression. HeLa cells were treated with Cisplatin or PAC1, or transfected with plasmids expressing HA-tagged 2B. The cells were assessed with the CCK-8 assay for cell viability. The values represented mean ± SD from triplicates of independent experiments and the viability of negative control cells without any treatment was set as 100%.

Other than HeLa cells, we also investigated the effect of 2B on KPNA1 in other cell types in our study. HT-29 is a human intestinal carcinoma cell line and RD is a human rhabdomyosarcoma cell line. Both cell lines are susceptible to EV-A71 infection. When HT-29 and RD cells were transfected with plasmids expressing 2B, cleavage of pro-caspase-3 occurred and KPNA1 degradation was observed in a dose-dependent manner in both cell types (Figures 5A,B), similar to the findings in HeLa cells, indicating that the effect of 2B on KPNA1 was not limited to HeLa cells.

We were interested in knowing whether active caspase-3, activated by stimuli other than viral 2B, was also able to degrade KPNA1, which appeared to be a target of caspase-3. Cisplatin, a potent apoptosis inducer and anticancer agent, and PAC1, an agonist of pro-caspase-3, were used to treat the cells as described (Putt et al., 2006; Peterson et al., 2009). As shown in Figure 5C, the treatment with both cisplatin and PAC1 activated pro-caspase-3, which was correlated with the cleavage of PARP, a known substrate of caspase-3, and KPNA1, an effect observed in the cells expressing viral 2B (Figure 5C). Cell viability was affected as well. While the treatment with Cisplatin and PAC1 caused evident cell death, expression of 2B protein led to a moderate decline of cell viability (Figure 5D).

### The N-Terminal Domain of 2B Was Responsible for the Induced Degradation of KPNA1

**FIGURE 6:**
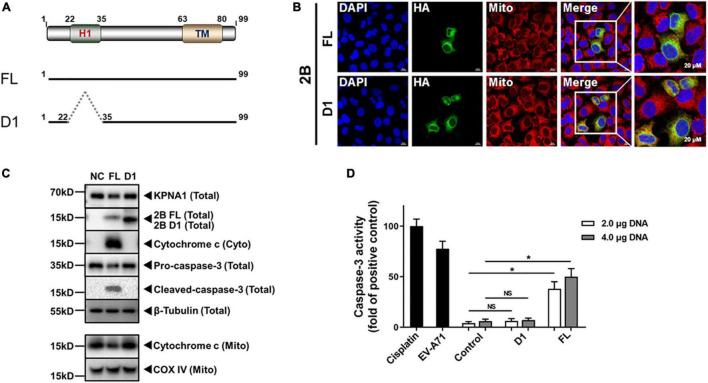
The N-terminal H1 domain of 2B appeared to be responsible for the induced degradation of KPNA1. **(A)** Schematic illustration of full-length (FL) and truncated constructs (D1) of EV-A71 2B. **(B)** Subcellular localization of full-length and truncated D1 (△22–35) 2B in cells. HeLa cells on glass slides were transfected with plasmids expressing HA-tagged full-length or truncated D1 constructs (2.0 μg) for confocal immunofluorescence imaging with staining of antibodies for HA tag. DAPI staining was for visualizing the nuclei. Mitochondria were labeled with MitoTracker™ Deep Red FM. Magnification, × 630. **(C)** Effect of the D1 deletion mutant on the release of cytochrome c and degradation of KPNA1. HeLa cells were transfected with plasmids expressing HA-tagged full length or D1 mutant (2.0 μg). Whole cell lysates and the mitochondrial (Mito) and cytosolic (Cyto) fractions were prepared at 36 h post transfection for western blot analyses with antibodies for KPNA1, HA tag, cytochrome *c*, pro-caspase-3, cleaved-caspase-3, COX IV, or β-Tubulin. **(D)** Effect of D1 deletion on caspase-3 enzymatic activity. HeLa cells were either infected with EV-A71 or transfected with plasmids expressing full length or D1 mutant 2B (2.0/4.0 μg). HeLa cells were treated with Cisplatin (30 μM) as positive control. Supernatants of the cell lysates were prepared 36 h later from the cells with different treatments for measuring the enzymatic activity of caspase-3 using Ac-DEVD-pNA as a substrate. The caspase-3 activity in Cisplatin-treated cells was set as 100%. *t* tests were conducted between control groups and transfected groups based on the same amount of plasmid DNA used for transfection. NS, no significance; *P < 0.05.

Structurally EV-A71 2B possesses a proposed N-terminal hydrophilic helix (H1, aa 22–35), an amphipathic α-helix domain (aa 37–55), and a putative C-terminal hydrophobic α-helix or transmembrane region (TM, aa 63–80) (Cong et al., 2016). To map the domain of 2B which may be involved in inducing the degradation of KPNA1, we generated one deletion mutant, named as D1, as shown in Figure 6A. HeLa cells were transfected with plasmids expressing a full length (FL) or deletion mutant (D1) 2B and subcellular localization of the truncated mutant was determined under a confocal microscope. As shown in Figure 6B, the truncated D1 2B protein, lacking in H1 domain, was localized close to the mitochondrial region in the cytosol, similar to the full length 2B.

We next examined how the deletion affected the reduction of KPNA1 levels in cells expressing the truncated 2B mutant. As shown in Figure 6C, degradation of KPNA1 was induced in the presence of FL 2B, but the level of KPNA1 remained unchanged in the presence of D1 2B, suggesting that the H1 (aa 22–35) domain of EV-A71 2B appeared to be involved in the caspase-3 dependent degradation of KPNA1. In addition, we detected the cytochrome *c* release and pro-caspase-3 cleavage in the cells expressing FL, but not D1 2B, indicating that viral 2B could induce a change of the mitochondrial membrane potential for the release of cytochrome c, which may be negatively affected by the H1 domain.

We also confirmed that caspase-3 facilitated the degradation of KPNA1 through its proteinase activity. Enzymatic activities of caspase-3 were quantified in the presence of the truncated 2B mutant in comparison with FL 2B. Cell extracts were prepared from the cells expressing D1 or FL 2B for measuring relative caspase-3 activities. Cisplatin was used to stimulate the cells as a positive control in this assay. As shown in Figure 6D, the relative caspase-3 activity in EV-A71-infected cells was close to the level of the positive control, and moderate increases of the caspase-3 activity were detected in the cells expressing FL 2B. However, little or no caspase-3 activity was detected in the cells expressing D1 2B, indicating that the H1 domain of the viral 2B may play a key role in activating pro-caspase-3, leading to degradation of KPNA1 in EV-A71-infected cells.

### Increased Expression of Viral 2B Protein in Cells Infected With EV-A71

**FIGURE 7:**
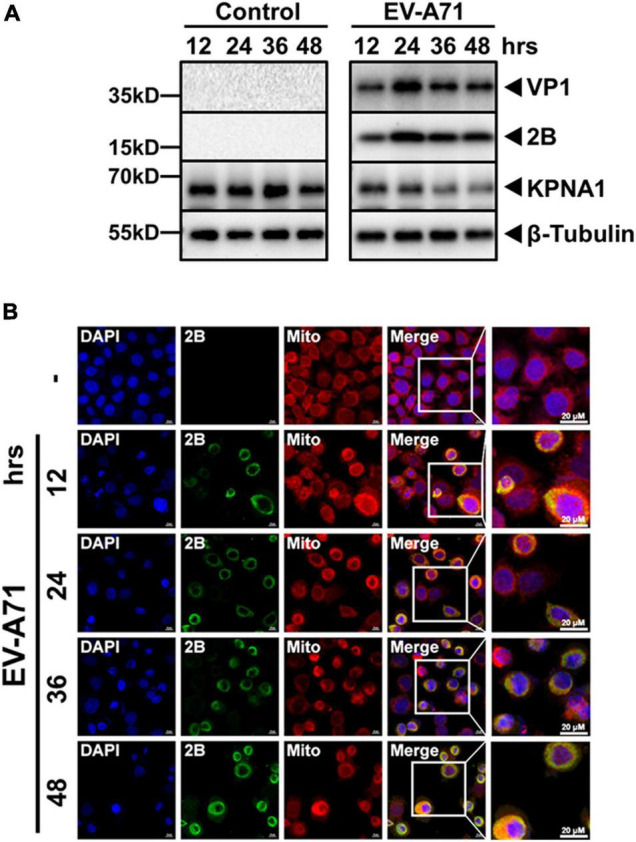
Expression and localization of viral 2B protein in EV-A71 infected cells. HeLa cells were infected with EV-A71 at an MOI of 1. Cell lysates were prepared at indicated time points p.i for western blot analyses with antibodies for VP1, 2B, KPNA1 or β-Tubulin **(A)**. The cells, infected or non-infected, were fixed at indicated time points p.i. and subjected to confocal immunofluorescence imaging with antibodies for viral 2B **(B)**. Cell nuclei were stained with DAPI and the mitochondria were stained with MitoTracker™ Deep Red FM. Magnification, × 630.

The expression of viral 2B in EV-A71-infected cells was examined in this study. Viral 2B could be detected at 12 h p.i. in EV-A71-infected cells. The peak of 2B expression appeared to be around 24 h p.i., a pattern similar to viral VP1 as shown in the cell lysates (Figure 7A). On the other hand, the levels of KPNA1 started to decrease at 12 h and its degradation increased thereafter over time, which matched the expression dynamics of viral 2B. In infected cells, viral 2B was localized near the mitochondrial region in the cytoplasm, which was similar to exogenously expressed FL or D1 2B mutant as shown under a confocal microscope (Figure 7B).

### Decreased Degradation and Viral Replication in the Cells Infected With Recombinant EV-A71 Deficient of the 2B H1 Domain

To characterize the biological function of the 2B H1 domain in the degradation of KPNA1 in EV-A71-infected cells, we constructed recombinant EV-A71 virus deficient of the H1 domain. To obtain the recombinant viruses, we first synthesized the cDNA of the full length viral genome. The cDNA region of the H1 domain was subsequently removed to obtain an H1 deletion mutant of the genome. Viral RNA was prepared through an *in vitro* transcription system and the recombinant viruses were collected from the culture medium of the cells transfected with the infectious viral ssRNA according to a strategy as previously reported (Tan et al., 2016). Two viable recombinant virus strains were obtained, which included the one with the full length genome of the Fuyang 0805 strain, rFY EV-A71, and another with a truncated genome deficient of the H1 domain, rD1 EV-A71. Subsequent Sanger sequencing confirmed that there were no extra mutations in the recombinant viral genome sequences of both rFY and rD1 EV-A71.

**FIGURE 8:**
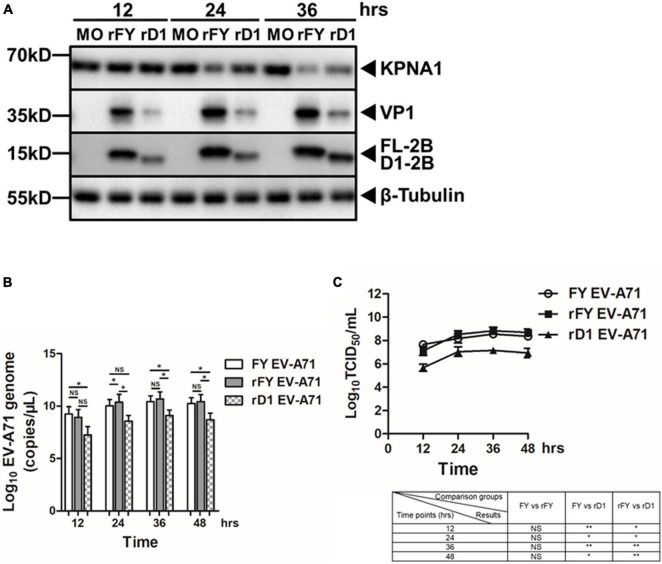
Decreased KPNA1 degradation and viral replication in rD1 EV-A71-infected cells. **(A)** Decreased degradation of KPNA1. HeLa cells were infected with rFY or rD1 EV-A71 at an MOI of 1. The cell lysates were prepared at indicated time points p.i. for western blot analyses with antibodies for KPNA1, VP1, 2B, or β-Tubulin. **(B,C)** Effect of 2B deficient of the H1 domain on viral replication. HeLa cells were infected with either rFY or rD1 EV-A71. Total RNA was prepared at various time points p.i. for real-time RT-PCR to measure viral RNA copy numbers **(B)**; Cell culture medium was sampled at various time points p.i. for infectious virus titration **(C)**. Error bars represent standard deviations of the results of three repeated experiments. NS, no significance; *P < 0.05; and **P < 0.01.

We infected HeLa cells with recombinant rFY or rD1 EV-A71 at an MOI of 1 and cell lysates were prepared at indicated time points p.i. for western blot analyses. As shown in Figure 8A, the levels of KPNA1 decreased significantly at 24 and 36 h p.i. in rFY-infected cells. However, the degradation of KPNA1 was prevented to certain degrees at 24 and 36 h p.i. in rD1-infected cells, suggesting that the H1 domain of 2B played a key role in the stability of KPNA1 in EV-A71-infected cells. On the other hand, we observed that the levels of VP1 and 2B proteins decreased as well in rD1 EV-A71-infected cells, indicating that virus replication may be affected negatively when the H1 domain of 2B was removed from the virus genome.

We quantified viral RNA copy numbers and titrated infectious viral titers in the cells infected with both native and recombinant viruses. Total RNA was prepared from the infected cells for real-time RT-PCR at various time points p.i. As shown in Figure 8B, viral RNA copy numbers appeared to be comparable in native or recombinant FY EV-A71-infected cells. However, the viral RNA copy numbers were significantly lower in recombinant rD1 EV-A71-infected cells. Culture medium was collected for titration of infectious viruses by a standard TCID_50_ assay. While the infectious viral titers were similar between the cells infected with the native and rFY EV-A71, the viral titers in the cells infected with rD1 EV-A71 were significantly lower (Figure 8C), indicating that the rD1 EV-A71 replication appeared to be inhibited with 2B deficient of the H1 domain.

### Increased Sensitivity of rD1 EV-A71 to IFN-α in Infected Cells

**FIGURE 9:**
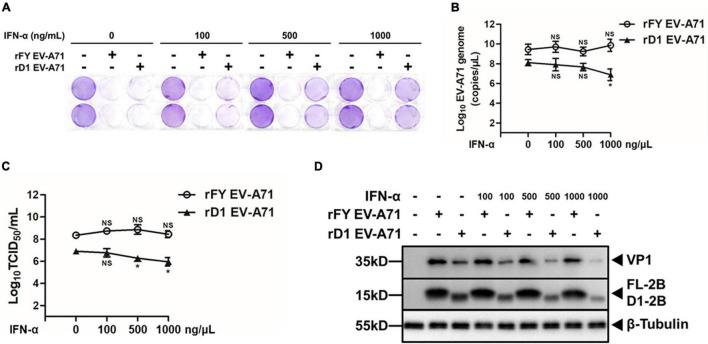
Increased sensitivity of rD1 EV-A71 to IFN-α in infected cells. HeLa cells were pre-treated with IFN-α for 2 h, followed by infection with rFY or rD1 EV-A71 at an MOI of 1. **(A)** Cytopathic effects of infected cells pre-treated with IFN-α. Cells were fixed at 48 h p.i. and stained with 0.5% crystal violet. Darker crystal violet staining indicates more viable cells present after infection. **(B–D)** Decreased viral replication in rD1 EV-A71-infected cells treated with IFN-α. Total RNA was prepared from the infected cells, pre-treated with IFN-α, at 48 h p.i. for real-time RT-PCR to measure viral RNA copy numbers with the primers specific for VP1 gene **(B)**. Culture medium from the infected cells, pre-treated with IFN-α, were sampled at 48 h p.i. and infectious viral titers (TCID_50_) were determined **(C)**. Error bars represented standard deviations of the results for three repeated experiments. Cell lysates were prepared from the infected cells, pre-treated with IFN-α, at 48 h p.i. for western blot analyses with antibodies for VP1, 2B, or β-Tubulin **(D)**.

The sensitivity of the mutated rD1 EV-A71 to IFN-α in infected cells was evaluated. HeLa cells were pre-treated with IFN-α at varying concentrations for 2 h prior to infection with either rFY or rD1 EV-A71 (MOI = 1). Cytopathic effect (CPE) was observed under a light microscope after a crystal violet staining of the cells at 48 h p.i. As shown in Figure 9A, the monolayers of rFY EV-A71-infected cells were wiped out even with increasing doses of IFN-α were added in the cultures, a sign of insensitivity of rFY EV-A71 to the antiviral effect of IFN-α. However, in the monolayers infected with rD1 EV-A71, more cells survived in the presence of higher concentrations of IFN-α, demonstrating that rD1 EV-A71 was more sensitive to IFN-α and the cell death caused by rD1 EV-A71 could be effectively inhibited by the treatment of IFN-α in a dose-dependent manner (Figure 9A).

Viral RNA copy numbers were measured in the infected cells pre-treated with IFN-α. As shown in Figure 9B, viral RNA copy numbers decreased and the replication of rD1 EV-A71 was inhibited in the presence of higher IFN-α concentrations, whereas the replication of rFY EV-A71 was not suppressed by IFN-α (Figure 9B). Correspondingly, we observed that the infectious virus titers decreased in rD1 EV-A71-infected cells, pre-treated with IFN-α (Figure 9C). Cell lysates were prepared from the infected cells with either rFY or rD1 EV-A71 for western blot analyses, which showed a lower expression of viral VP1 and 2B in rD1 EV-A71-infected cells pre-treated with IFN-α (Figure 9D). These data demonstrate that the N-terminal (H1) domain of 2B may play a critical role in disrupting the type I IFN signaling in EV-A71-infected cells, which could be one of the strategies for EV-A71 to evade host innate immunity.

### Compromised Inhibitory Effect on Type I Interferon Responses in rD1 EV-A71 Infection

**FIGURE 10:**
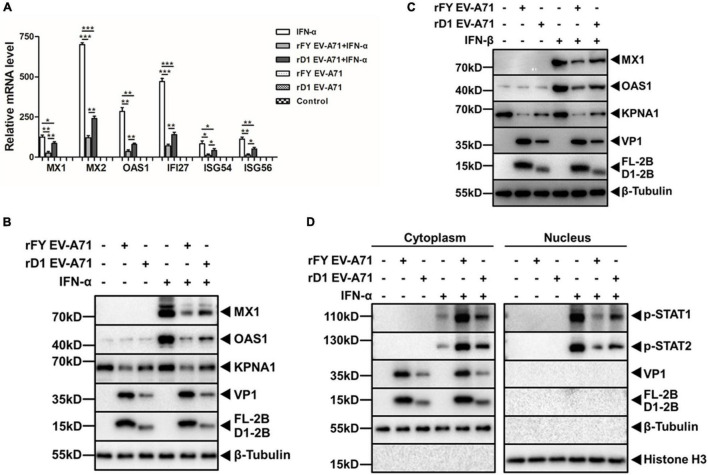
Decreased inhibitory effect on type I IFN response stimulated by IFN-α/β in rD1 EV-A71-infected cells. **(A)** Induction of ISGs in rD1-infected cells. HeLa cells were infected with rFY or rD1 EV-A71 for 24 h, followed by treatment with or without IFN-α (25 ng/μL) for another 2 h. Total RNA was prepared from the cells for real-time RT-PCR to measure relative levels of the ISG gene transcripts. Values represented mean ± SD from triplicates of independent experiments. *t* tests were conducted on values from different groups. *P<0.05; **P<0.01; ***P<0.001. **(B,C)** Decreased reduction of ISGs and KPNA1 in rD1-infected cells. HeLa cells were infected with rFY or rD1 EV-A71 for 24 h, followed by treatment with or without IFN-α **(B)** or IFN-β **(C)** (25 ng/μL) for another 2 h. Cell lysates were prepared for western blot analyses with antibodies for MX1, OAS1, or KPNA1. **(D)** Increased translocation of p-STAT1/2 into the nucleus in rD1-infected cells. HeLa cells were infected with rFY or rD1 EV-A71 for 24 h, followed by stimulation with IFN-α (25 ng/μL) for another 30 min. The cytosolic and nuclear fractions were prepared from the cells for western blot analyses with antibodies for p-STAT1 or p-STAT2. β-tubulin and histone H3 were detected as loading controls for cytosolic and nuclear fractions, respectively.

To further confirm that the H1 domain of 2B was involved in the type I IFN signaling, HeLa cells were infected with rFY or rD1 EV-A71, followed by the treatment with IFN-α to activate JAK-STAT signaling. We measured the transcriptional upregulation of ISGs in the cells p.i. by real-time RT-PCR and found that rFY EV-A71 inhibited the IFN-α-induced mRNA upregulation of ISGs including MX1, MX2, OAS1, IFI27, ISG54, and ISG56. However, the inhibition of the ISGs was reversed in general in the cells infected with rD1 EV-A71 (Figure 10A). Inhibition of MX1 and OAS1 was also exhibited at the protein level when the cell lysates from the IFN-α-treated cells, infected with rFY or rD1 EV-A71, were analyzed by western blot analyses. As shown in Figure 10B, the inhibition of MX1 and OAS1 was reversed in the cells infected with rD1, as compared to the cells infected with rFY EV-A71. We also tested IFN-β, another type I IFN, for its treatment of the cells infected with either rFY or rD1 EV-A71, and observed the reversed inhibition of MX1 and OAS1 in rD1 EV-A71-infected cells as well (Figure 10C).

The translocation of p-STAT1 in rFY or rD1 EV-A71-infected cells, pre-treated with IFN-α, was further analyzed by western blot analyses. As shown in Figure 10D, p-STAT1/2 was blocked effectively from translocation into the nucleus in the rFY EV-A71-infected cells stimulated by IFN-α. However, a significant amount of p-STAT1/2 was translocated into the nucleus in rD1 EV-A71-infected cells stimulated by IFN-α (Figure 10D), suggesting that the blockage of p-STAT1/2 from translocating into the nucleus was incomplete and IFN-α signaling was not suppressed effectively in the cells infected with rD1 EV-A71. In another word, in rD1 EV-A71-infected cells, KPNA1 may remain intact without much degradation and be capable of forming the ISGF3 complexes with p-STAT1/2 and carrying them into the nucleus in the cells infected by EV-A71 with 2B deficient of the H1 domain.

## Discussion

EV-A71 has been known to be resistant to antiviral activities induced by type I IFN, which is critical in viral pathogenesis. Studies have been performed to elucidate the mechanisms about how the viral resistance to the IFN response occurs during EV-A71 infection. Our previous study showed that EV-A71 suppressed the IFN responses by blocking JAK/STAT signaling through inducing KPNA1 degradation (Wang C. et al., 2017). It appeared that caspase-3 activity was involved in degrading KPNA1 but the question remained on how caspase-3 was activated and whether any EV-A71 viral proteins could play a role in the degradation of KPNA1. In this study, we demonstrated that, among all the EV-A71 proteins, 2B was the one involved in the degradation of KPNA1, which played a critical role in the inhibition of the JAK/STAT signaling pathway. Our data showed that the N-terminal H1 domain of viral 2B participated in the caspase-3 dependent degradation of KPNA1. We confirmed the interaction of p-STAT1 with KPNA1 upon the type I IFN stimulation as previous described and found that the amount of p-STAT1-KPNA1 complex decreased in the cells expressing viral 2B after IFN-α stimulation. Although viral 2B did not interact with KPNA1 or p-STAT1 directly, decreased levels of KPNA1 led to a lowered amount of the p-STAT1-KPNA1 complex, which may result in the blockage of the complex translocation into the nucleus to activate the transcription of ISGs upon IFN-α stimulation.

The mechanism of the KPNA1 degradation induced by viral 2B was further explored in this study. Our data showed that KPNA1 decreased post-transcriptionally, mediated by caspase-3, while the activation of pro-caspase-3 was dependent on viral 2B. In EV-A71-infected cells, viral 2B was localized in the mitochondrial area in the cytoplasm (Figure 7B), which was in conformity with the results from a previous study that EV-A71 2B was localized to the mitochondria and enhanced the permeability of the outer membrane through interacting with pro-apoptotic protein Bax (Cong et al., 2016). We have observed the increased level of cytochrome c in the cytosol in the cells expressing viral 2B, which probably led to the activation of pro-caspase-3 (Figure 6C). Structurally, 2B of enteroviruses is putatively composed of an amphipathic α-helix domain, a transmembrane region, and a C-terminal hydrophobic α-helix (vanKuppeveld et al., 1996; van Kuppeveld et al., 2005). A “barrel-stave model” has been proposed for a functional 2B structure, in which four 2B monomers are oligomerized to form a tetramer which exposes the hydrophobic faces to the lipid bilayer and the hydrophilic faces toward each other to form an aqueous pore in membrane structures (Shai, 1999; Nieva et al., 2003). In addition to the hydrophobic domains which are common in enterovirus 2B, an extra N-terminal hydrophilic helix (aa 22–35, named as H1) was proposed, which was shown to be critical to the release of cytochrome c, while its transmembrane region (TM) was responsible for targeting mitochondrial membrane (Cong et al., 2016). Interestingly, in this report we confirmed that the H1 domain appeared to be important in the release of cytochrome c from the mitochondria to the cytosol and activation of pro-caspase-3. Therefore, we hypothesize that the elements in the H1 domain of viral 2B were essential to the caspase-3 dependent degradation of KPNA1 and resistance of EV-A71 to the type I IFN signaling.

Enterovirus 2B proteins may function by interacting with cellular membranes as a mechanism for their interaction with the cell. Viral 2B of both poliovirus and Coxsackie B3 virus targets endoplasmic reticulum and Golgi apparatus and directly facilitates contents releasing, especially Ca^2+^, which plays a critical role in the virus life cycle by inducing a series of cytotoxic reactions to promote virus replication and release (Aldabe et al., 1997; vanKuppeveld et al., 1997; Agirre et al., 2002; de Jong et al., 2003, 2008; Campanella et al., 2004; Patargias et al., 2009). The 2B proteins of these enteroviruses could enhance the permeability of organelle membranes and inhibit protein trafficking through interacting with innate membrane proteins, which in turn facilitates accumulation of vesicles in the cytosol (de Jong et al., 2003; Patargias et al., 2009; Zhou et al., 2009). These vesicles tend to clump together, where various virus proteins and viral genomic RNA involved in viral replication are adherent to surface to form a complex called the replication factory, essential for propagation of enteroviruses (Bienz et al., 1994; Egger et al., 2000). Our results in this study, that viral 2B may interact with the mitochondria through its H1 domain to promote cytochrome c release and caspase-3 activation, leading to KPNA1 degradation, demonstrate that enterovirus 2B does possess multiple functions. 2B could act directly to impact on the replication of enteroviruses. A recent report showed that EV-A71 2B interacted with transcription factor ILF2 in the cytoplasm to inhibit its negative regulation on virus RNA replication, suggesting that viral 2B could modulate EV-A71 replication in a direct manner (Jin et al., 2020).

The mechanism of viral 2B protein to assist EV-A71 to evade the type I IFN-mediated broad-spectrum antiviral effects through promoting KPNA1 degradation, probably *via* its N-terminal H1 domain, is one of many strategies used by EV-A71 during its infection. EV-A71 employs various means to antagonize the JAK/STAT and other signaling pathways and thwart host innate immunity, which ensures its virulence in susceptible hosts and expands its invasiveness in the central nervous system beyond the initial oral and gastrointestinal infection. Previous studies have demonstrated a variety of strategies used by EV-A71 to inhibit the type I anti-viral IFN responses. Non-structural proteins, 2A and 3C, of EV-A71 possess protease activities, which play diverse and important roles in dysregulating innate immunity by degrading multiple important host factors that are critical in IFN signaling pathways (Lei et al., 2010, 2011, 2013; Wang B. et al., 2013). However, KPNA1 was not the target of either 2A or 3C as shown in this study. Instead, our data demonstrate that 2B may interact with the mitochondrial membrane and indirectly degrade KPNA1, resulting in the modulation of the JAK/STAT pathway. How viral 2B of EV-A71 affects the permeability of the mitochondrial membrane through its H1 domain remains a question and warrants further investigation, which will help fully unveil the molecular mechanism of EV-A71 resistance to the IFN response in infected cells and patients.

## Data Availability Statement

The raw data supporting the conclusions of this article will be made available by the authors, without undue reservation.

## Author Contributions

MS, YJ, and ZX conceived and coordinated the study. MS, QL, JX, and CW designed, performed, and analyzed the experiments. KY and ZL provided reagents, technical assistance, and contributed to completion of the studies. MS, CC, and ZX wrote the manuscript. All authors reviewed the results and approved the final version of the manuscript.

## Conflict of Interest

The authors declare that the research was conducted in the absence of any commercial or financial relationships that could be construed as a potential conflict of interest.

## Publisher’s Note

All claims expressed in this article are solely those of the authors and do not necessarily represent those of their affiliated organizations, or those of the publisher, the editors and the reviewers. Any product that may be evaluated in this article, or claim that may be made by its manufacturer, is not guaranteed or endorsed by the publisher.
